# Supplementary material for: Benzothiadiazole Affects Grape Polyphenol Metabolism and Wine Quality in Two Greek Cultivars: Effects during Ripening Period over Two Years
Source: Plants (Basel). 2023 Mar 4;12(5):1179. doi: 10.3390/plants12051179 (PMC10005230; doi:10.3390/plants12051179)
Supplement: Supplementary file 1 [file plants-12-01179-s001.zip › plants-2243817-supplementary.pdf]

plants

MDPI

Article,

Benzothiadiazole affects grape polyphenol metabolism and wine quality in two Greek cultivars: Effects during ripening period over two years.

Dimitrios- Evangelos Miliordos[1,2,3,*], Anastasios Alatzas[2], Nikolaos Kontoudakis[1,4] , Marianne Unlubayir[3], Polydefkis Hatzopoulos[2], Arnaud Lanoue[3*] and Yorgos Kotseridis[1]

1 Laboratory of Oenology and Alcoholic Beverage Drinks, Department of Food Science and Human Nutrition, Agricultural University of Athens, 75 Iera Odos, 11855 Athens, Greece
2 Molecular Biology Laboratory, Department of Biotechnology, Agricultural University of Athens, 75 Iera Odos, 11855 Athens, Greece
3 EA 2106 Biomolécules et Biotechnologie Végétales, UFR des Sciences Pharmaceutiques, Université de Tours, 31 Av. Monge, F37200 Tours, France
4 Department of Agricultural Biotechnology and Oenology, International Hellenic University, 1st km Drama-Mikrochori, 66100 Drama, Greece

* Correspondence: dim.miliordos@gmail.com (D.E.M); arnaud.lanoue@univ-tours.fr (A.L.)

Citation: Miliordos, D.E.; Alatzas, A.; Kontoudakis, N.; Unlubayir, M.; Hatzopoulos, P.; Lanoue, A.; Kotseridis, Y..

Academic Editor: Firstname Lastname

Received: date
Revised: date
Accepted: date
Published: date

Copyright: © 2023 by the authors. Submitted for possible open access publication under the terms and conditions of the Creative Commons Attribution (CC BY) license (https://creativecommons.org/licenses/by/4.0/).

Table S1: Date of biostimulant applications and samplind dates during 2019 and 2020 vintages.

	Biostimulant Application			
	Mouhtaro		Savvatiano	
	2019	2020	2019	2020
1st application	16th July	17th July	16th August	21th August
2nd application	23nd July	24th July	23st August	27th August
3rd application	30th July	31st August	30th August	4th September
	Sampling dates			
1st Sampling (Veraison)	30th July	31st August	30th August	4th September
2nd Sampling (Mid Veraison)	15th August	18th August	10th September	15th September
3rd Sampling (Harvest)	3th September	8th September	29th September	24th September

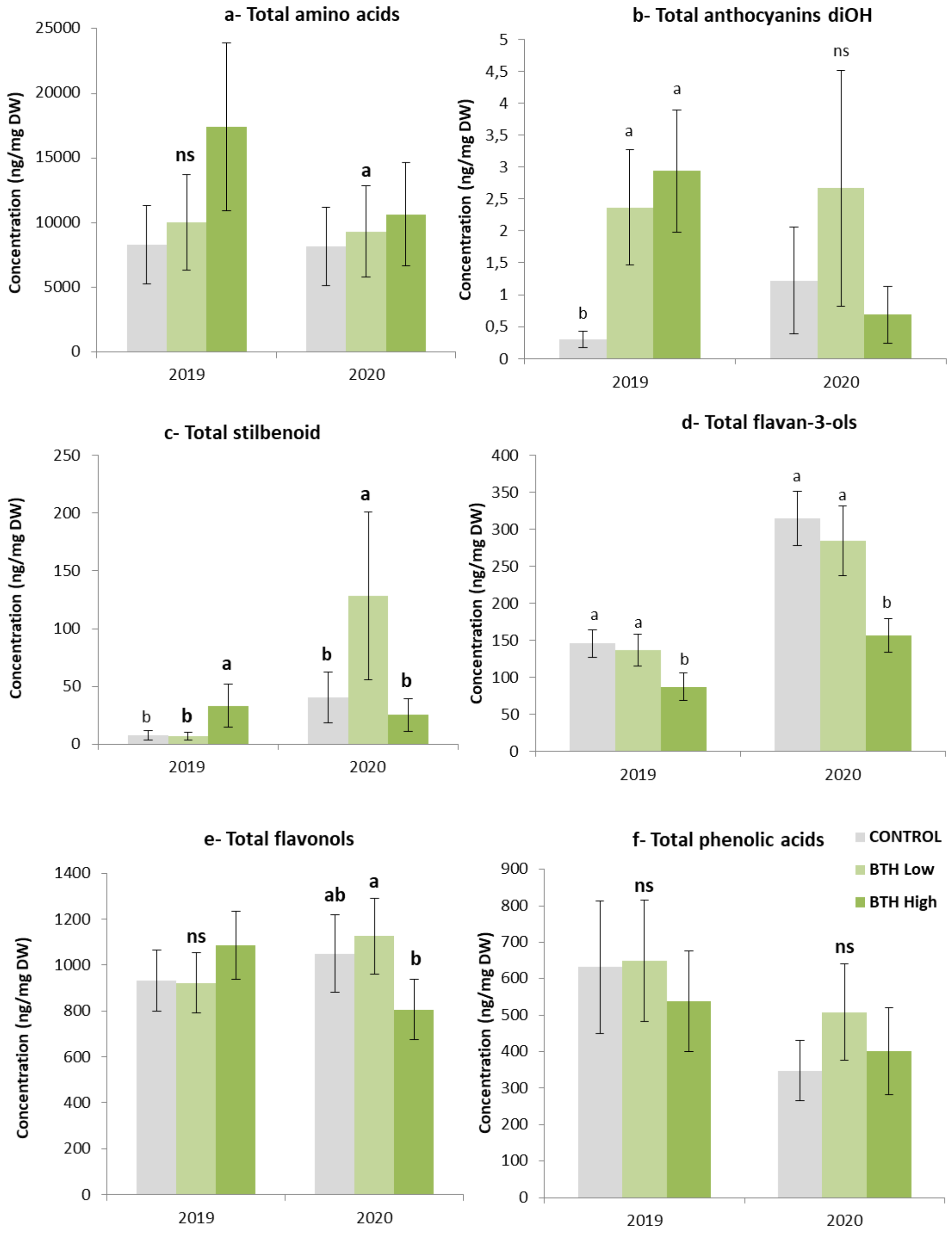

Figure S1. Total concentrations of amino acids (a), anthocyanins diOH (b), stilbenoids (c), flavan-3-ols (d), flavonols (e) and phenolic acids (f) in Savvatiano berries at Veraison (1st sampling) stage in 2019 and 2020 treated with benzothiadiazole. Control (grey), low concentration of benzothiadiazole (light green), and high concentration of benzothiadiazole (dark green). Error bars represent the standard deviations. Different letters indicate significant differences. No significant difference (ns) was found between values with the same letters (one-way ANOVA, p-value > 0.05).

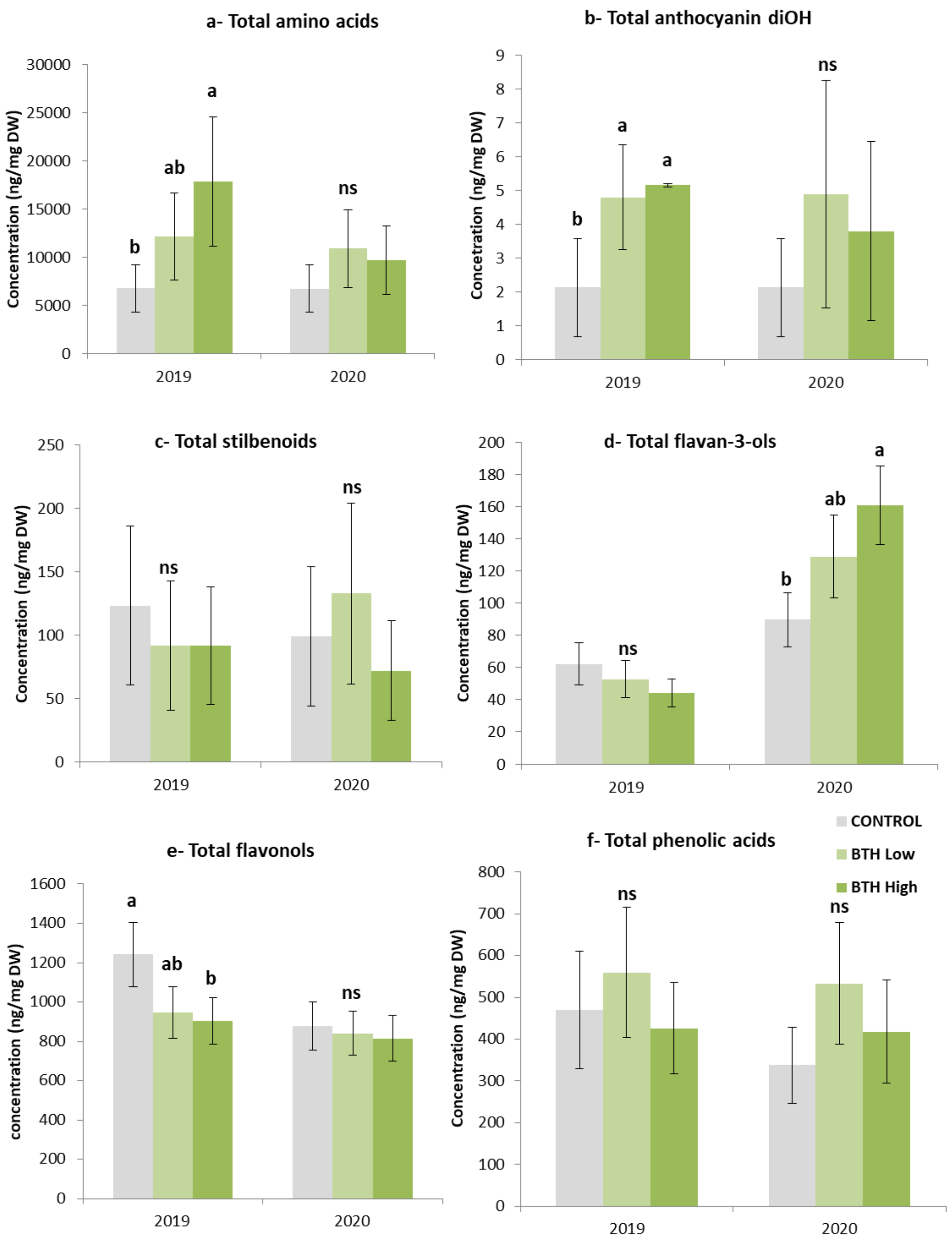

Figure S2. Total concentrations of amino acids (a), anthocyanins diOH (b), stilbenoids (c), flavan-3-ols (d), flavonols (e) and phenolic acids (f) in Savvatiano berries at Mid Veraison (2nd Sampling) stage in 2019 and 2020 treated with benzothiadiazole. Control (grey), low concentration of benzothiadiazole (light green), and high concentration of benzothiadiazole (dark green). Error bars represent the standard deviations. Different letters indicate significant differences. No significant difference (ns) was found between values with the same letters (one-way ANOVA, p-value > 0.05).

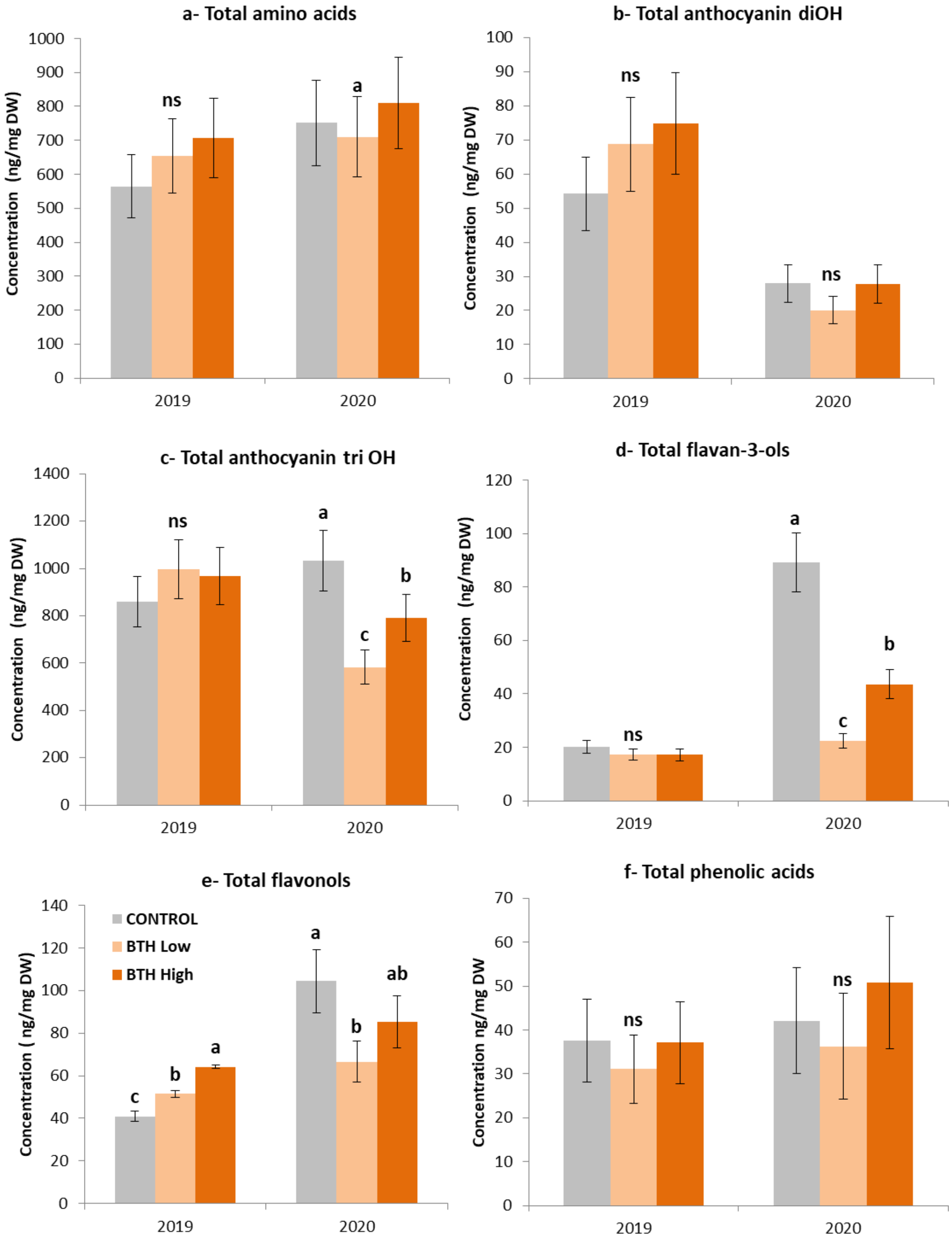

Figure S3. Total concentrations of amino acids (a), anthocyanins diOH (b), stilbenoids (c), flavan-3-ols (d), flavonols (e) and phenolic acids (f) in Mouhtaro berries at Veraison (1st sampling) stage in 2019 and 2020 treated with benzothiadiazole: control (grey), low concentration of benzothiadiazole (pale orange), and high concentration of benzothiadiazole (dark orange). Error bars represent the standard deviations. Different letters indicate significant differences. No significant difference (ns) was found between values with the same letters (one-way ANOVA, p-value > 0.05).

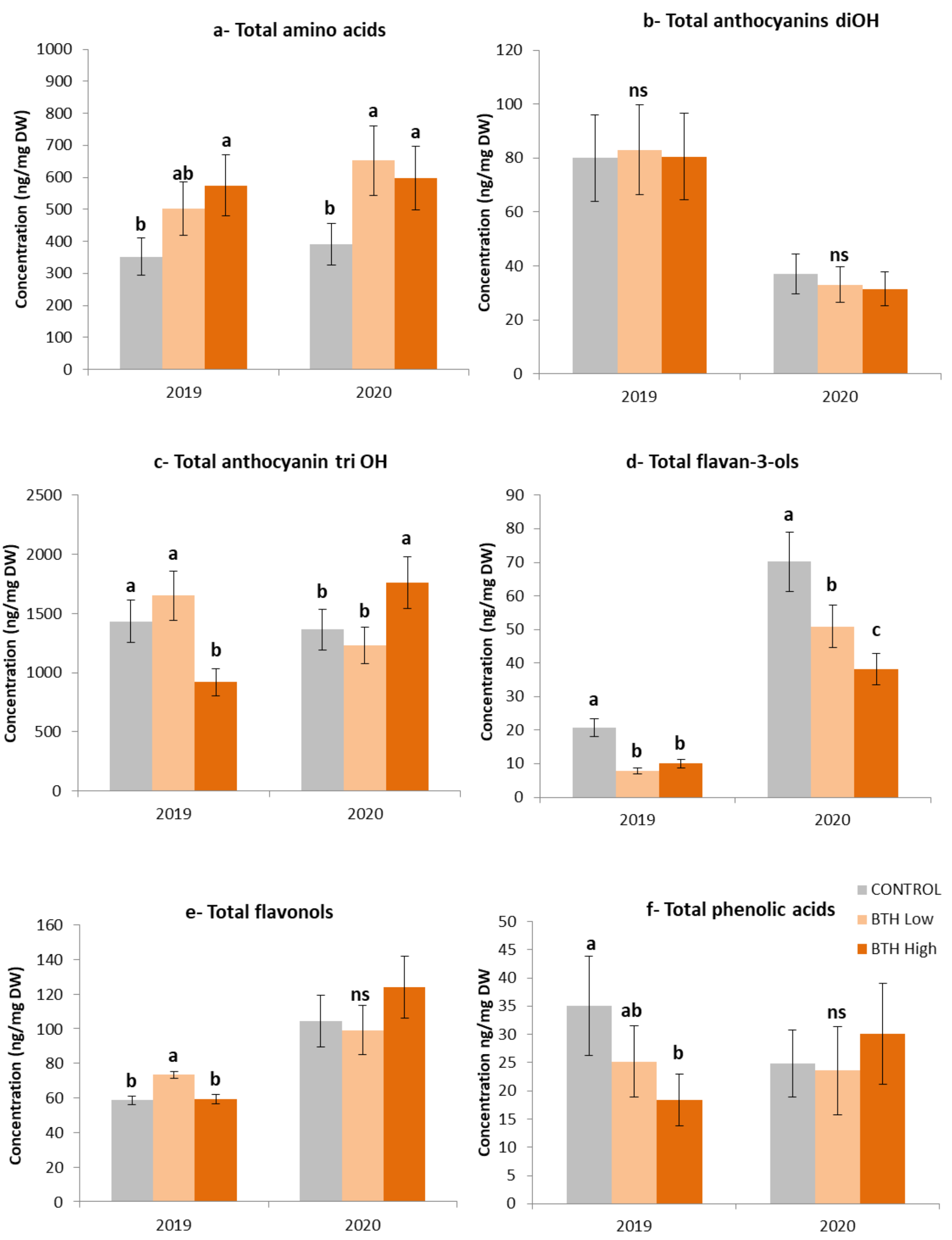

Figure S4. Total concentrations of amino acids (a), anthocyanins diOH (b), stilbenoids (c), flavan-3-ols (d), flavonols (e) and phenolic acids (f) in Mouhtaro berries at Mid Veraison (2nd sampling) stage in 2019 and 2020 treated with benzothiadiazole: control (grey), low concentration of benzothiadiazole (pale orange), and high concentration of benzothiadiazole (dark orange). Error bars represent the standard deviations. Different letters indicate significant differences. No significant difference (ns) was found between values with the same letters (one-way ANOVA, p-value > 0.05).

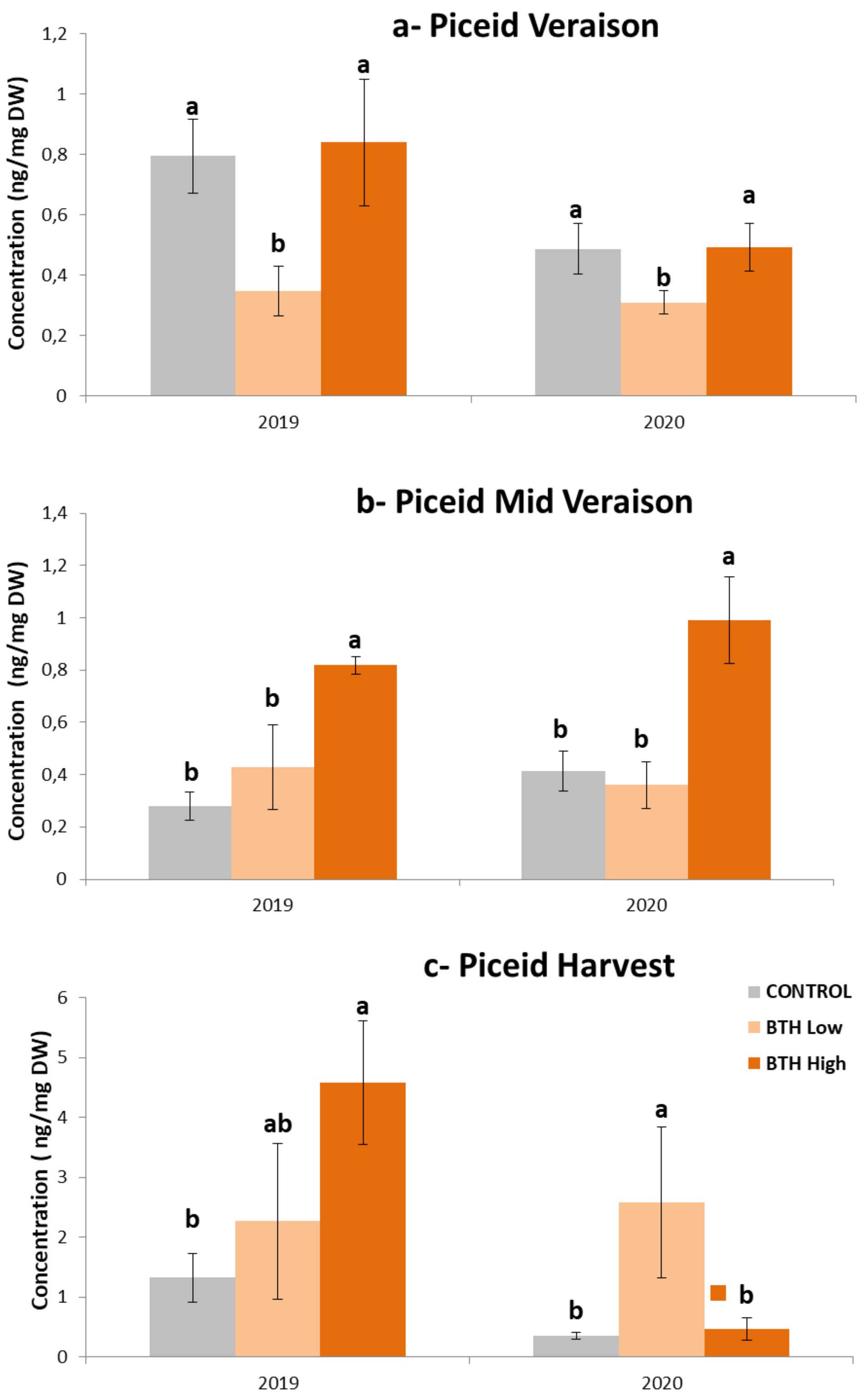

Figure S5. Concentrations of Piceid in Mouhtaro berries in three sampling dates, 2019 and 2020 treated with benzothiadiazole: control (grey), low concentration of benzothiadiazole (pale orange), and high concentration of benzothiadiazole (dark orange). Error bars represent the standard deviations. Different letters indicate significant differences. No significant difference (ns) was found between values with the same letters (one-way ANOVA. p-value > 0.05).

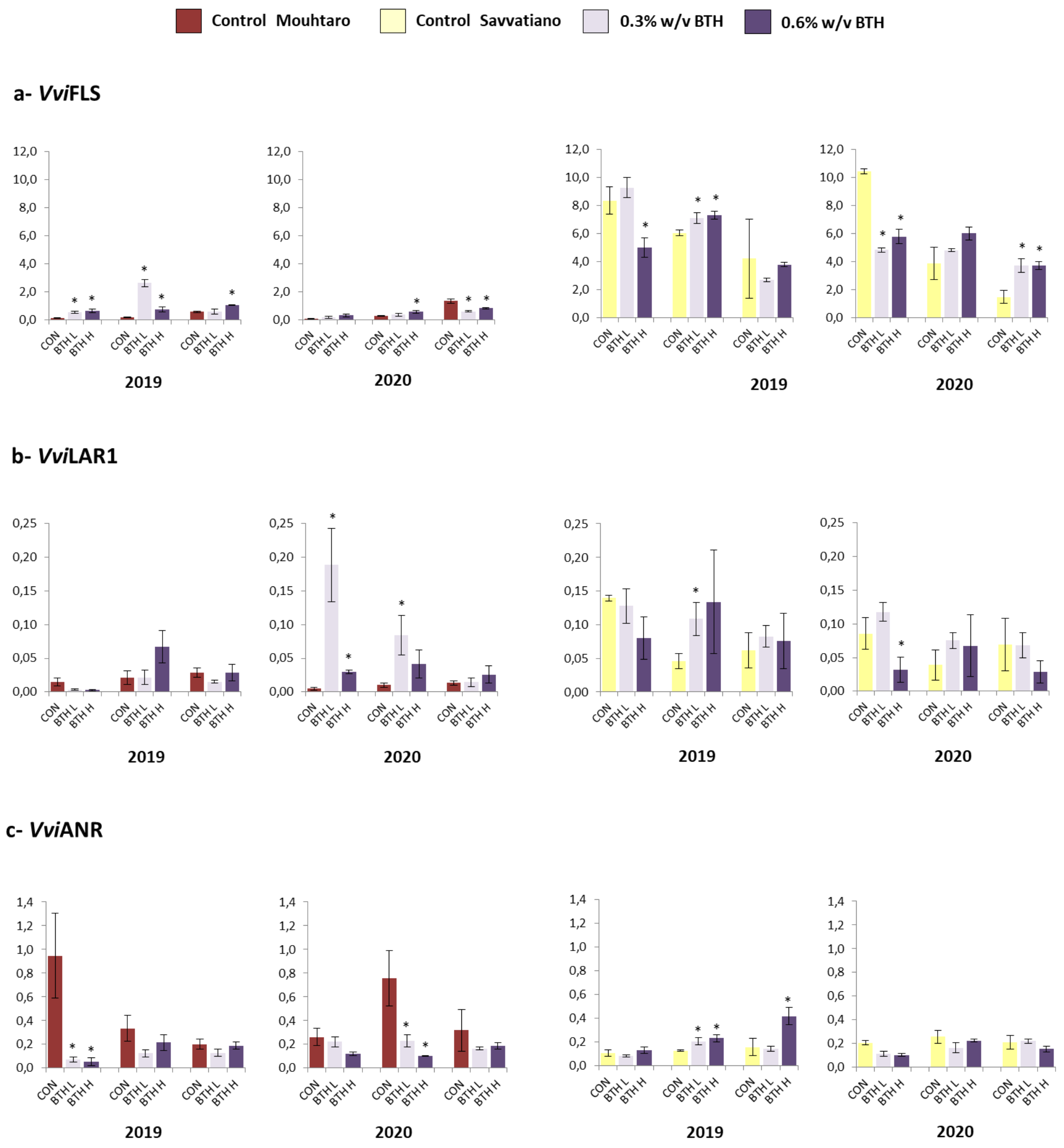

Figure S6. Expression level of genes involved in phenylpropanoid pathway: *Vvi*FLS (a), *Vvi*LAR1 (b) and *Vvi*ANR (c) in Mouhtaro and Savvatiano during two growing seasons (2019 and 2020). Vertical bars represent the standard deviation and asterisks indicate the statistically significant differences (Student's t-test. p–value < 0.05). The three sampling points (veraison; middle veraison. and harvest) are indicated under each graph.

Author Contributions: "Conceptualization, D.E.M., N.K. and A.L; methodology, D.E.M. and N.K software, D.E.M.; validation, D.E.M., A.A., N.K. M.U., A.L., P.H. and Y.K.; formal analysis, D.-E.M., N.K. and A.L.; investigation, D.E.M. and N.K.; resources, D.E.M., A.A., A.L. and N.K data curation, D.E.M.; writing—original draft preparation, D.E.M., A.A., N.K. and A.L; writing—review and editing, D.E.M., A.A., N.K. M.U., A.L., P.H. and Y.K.; visualization, D.E.M., A.A., A.L. and N.K.; supervision, D.E.M. and A.L. ; project administration, D.E.M. and A.L.; funding acquisition, Y.K. and D.E.M. All authors have read and agreed to the published version of the manuscript."

Funding: This research has been co-financed by the European Regional Development Fund of the European Union and Greek national funds through the Operational Program Competitiveness. Entrepreneurship and Innovation, under the call RESEARCH–CREATE– INNOVATE (project code: T1EDK-04200 MU-SA). This work was supported by the French Embassy in AthensGreece with the program "Programme de bourses pour séjour scientifique de haut niveau (SSHN) en France 2020–2021".

Data Availability Statement: The data presented in this study are available on request from the corresponding authors (pending privacy and ethical considerations).

Acknowledgments: The authors would like to thank oenologist Nikos Zacharias of the winery Muses Estate for providing the Savvatiano and Mouhtaro grapes

Conflicts of Interest: "The authors declare no conflict of interest."
